# *PKIB* involved in the metastasis and survival of osteosarcoma

**DOI:** 10.3389/fonc.2022.965838

**Published:** 2022-08-22

**Authors:** Rongxue Wan, Gu Yang, Qianzhen Liu, Xiaokang Fu, Zengping Liu, Huilai Miao, Huan Liu, Wenhua Huang

**Affiliations:** ^1^ Orthopaedic Center, Affiliated Hospital of Guangdong Medical University, Zhanjiang, China; ^2^ Guangdong Engineering Research Center for Translation of Medical 3D Printing Application, Guangdong Provincial Key Laboratory of Medical Biomechanics, School of Basic Medical Sciences, Southern Medical University, Guangzhou, China; ^3^ Guangdong Innovation Platform for Translation of 3D Printing Application, Southern Medical University, The Third Affiliated Hospital of Southern Medical University, Southern Medical University, Guangzhou, China; ^4^ Department of Hepatobiliary Surgery, Affiliated Hospital of Guangdong Medical University, Zhanjiang, China; ^5^ The Key Laboratory of Diagnosis and Repair in Liver Injury, Guangdong Medical University, Zhanjiang, China; ^6^ Department of Orthopedics, Affiliated Traditional Chinese Medicine Hospital, Southwest Medical University, Luzhou, China; ^7^ National Traditional Chinese Medicine Clinical Research Base, The Affiliated Traditional Chinese Medicine Hospital of Southwest Medical University, Luzhou, China

**Keywords:** PKIB, metastasis, AIM2, osteosarcoma, Akt, nomogram

## Abstract

Osteosarcoma is frequently metastasized at the time of diagnosis in patients. However, the underlying mechanism of osteosarcoma metastasis remains poorly understood. In this study, we evaluated DNA methylation profiles combined with gene expression profiles of 21 patients with metastatic osteosarcoma and 64 patients with non-metastatic osteosarcoma from TARGET database and identified *PKIB* and *AIM2* as hub genes related to the metastasis of osteosarcoma. To verify the effects of *PKIB* on migration and invasion of osteosarcoma, we performed wound-healing assay and transwell assay. The results showed that *PKIB* significantly inhibited the migration and invasion of osteosarcoma cells, and the Western blot experiments showed that the protein level of E-cad was upregulated and of VIM was downregulated in 143-B cell recombinant expression PKIB. These results indicate that PKIB inhibit the metastasis of osteosarcoma. CCK-8 assay results showed that PKIB promote the proliferation of osteosarcoma. In addition, the Western blot results showed that the phosphorylation level of Akt was upregulated in 143-B cells overexpressing PKIB, indicating that PKIB promotes the proliferation of osteosarcoma probably through signaling pathway that Akt involved in. These results give us clues that PKIB was a potential target for osteosarcoma therapy. Furthermore, combined clinical profiles analysis showed that the expression of *AIM2*- and *PKIB*- related risk scores was significantly related to the overall survival of patients with osteosarcoma. Thus, we constructed a nomogram based on *AIM2* and *PKIB* expression–related risk scores for osteosarcoma prognostic assessment to predict the 1-, 2-, 3-, and 5-year overall survival rate of patients with metastatic osteosarcoma, assisting clinicians in the diagnosis and treatment of metastatic osteosarcoma.

## Introduction

Osteosarcoma is a primary malignant bone tumor that mainly threatens the life of children and adolescents (1, 2). Available treatments include amputation or limb-sparing surgeries, as well as chemo- and immunotherapies (3). However, the overall response rate is low, and patients often develop metastasis and relapse. It was reported that about 15% of patients with osteosarcoma had metastatic cancer when diagnosed, with lung and bone being the two most affected organs by metastasis (4). Despite being toxic to the kidney and liver, chemotherapy has improved overall 2-year survival rates of patients with osteosarcoma from 17% to 66% (5, 6). Nevertheless, only 20% of patients with metastatic osteosarcoma survive for more than 5 years (7).

DNA methylation is an epigenetic modification of DNA that regulates the expression of genes through changes in chromatin structure, DNA conformation and stability, and by modulating interactions between DNA and proteins (8–12). DNA methylation was reported to be associated with tumorigenesis. Studies have shown that changes in DNA methylation of *WNT6* result in low expression of WNT, which is, in turn, associated with poor prognosis in children with osteosarcoma (13). It has also been reported that treatment with a DNA methylation inhibitor could upregulate the expression of miRNA-449c, consequently modulating the cell cycle and tumorigenesis potential of osteosarcoma cells (14). Several studies showed that DNA methylation is also associated with cancer metastasis progression. Oshima et al. (15) reported that altered DNA methylation profile of 14q32 miRNA cluster had an impact on metastasis development in the lung and liver in a preclinical model of metastatic cancer. Furthermore, transcription factors associated with proliferation and stemness of breast circulating tumor cells were found to be hypomethylated, which, in turn, supported metastasis progression (16). A study by Salomon et al. (17) also showed that DNA methylation could be responsible for the regulation of brain metastasis. Evidence suggests that DNA methylation may take part on osteosarcoma metastasis. Hypomethylation of the promoter of *IRX1*, leading to overexpression of IRX1, was reported in clinical osteosarcoma primary samples and osteosarcoma cell lines (18). Moreover, metastasis activity was found to be altered in osteosarcoma cell lines upon modulation of IRX1 expression (18). In addition, hypermethylation of the promoter of *APCDD1* was found to inhibit APCDD1 expression and to promote metastatic behavior of osteosarcoma cells (19). However, there is no available model to study the systemic relationship between DNA methylation and osteosarcoma metastasis, which may provide important clues to predict prognosis of patients with osteosarcoma.

Cancer metastasis is a complexity process; however, the systemic biology of cancer metastasis is still poorly understood (20). Epithelial-to-mesenchymal transition is a crucial process for cancer metastasis, in which epithelial cells obtained the phenotype of mesenchymal cells including decreased expression of adhesion molecular, such as E-cadherin (E-cad), on cell membrane, obtained vimentin (VIM)–rich intermediate filaments networks, and switched to the mesenchymal cells’ morphology (21, 22). It was reported that decreased expression of E-cad associated with the proliferation and metastasis of osteosarcoma (23). Some long non-coding RNAs and microRNAs were also reported related to osteosarcoma metastasis (24–26). However, the molecular mechanisms of osteosarcoma metastasis is still needed to uncover.

In our current study, we aimed to explore the mechanisms of metastasis of osteosarcoma through combining analysis approach. We identified that PKIB (protein kinase inhibitor-β) and AIM2 (absent in melanoma 2) were related to the metastasis and survival of osteosarcoma. In addition, PKIB promoted the proliferation of osteosarcoma cells probably through affecting phosphorylation level of Akt and inhibited the migration and invasion of osteosarcoma cells through affecting the expression of E-cad and VIM. All of our results showed that PKIB has potential as targets for osteosarcoma therapy, and the related nomogram provided a useful prognosis prediction model for osteosarcoma.

## Materials and methods

### Data collection

Expression and DNA methylation data of metastatic and non-metastatic osteosarcoma samples were downloaded from the TARGET database (https://ocg.cancer.gov/programs/target). The clinical features of the 86 patients with osteosarcoma included in the study are summarized in **Table 1**.

**Table 1:** Summary of patient demographics and characteristics.

Characteristics	Methylation Data	Expression Profile Data
**Gender**		
Male	47	45
Female	39	40
**Age**		
Median	15	15
Range	4–40	6–21
**Vital Status**		
Metastasis	22	21
Non-metastasis	64	64

### Development and validation of DNA methylation signature of osteosarcoma

The TARGET clinical cases were used as the training set to develop the risk score assessment model. Univariate analysis and log-rank test were used to identify methylation related genes with prognostic potential. P< 0.05 was used as reference to identify statistically valuable information. Cox proportional hazard model with a lasso penalty was used to find the best gene model through the R package “glmnet” (27). The best gene model was used to establish the *AIM2*/*PKIB* expression–based risk score, which was given by the following equation:


$$\text{Risk Score}(\text{RS})=\sum_{i=1}^{N}Exp_{i}*Coef_{i}$$

N is the representative number of prognostic mRNAs, *Exp*_*i*_ is the expression value of the mRNAs, and *Coef*_*i*_ is a single factor Cox regression coefficient. *Risk Score (RS)* is the multinode-weighted sum of risk scores.

### Survival analysis

The Kaplan–Meier (K-M) survival curves were generated to graphically demonstrate the overall survival (OS) of the high-risk group and low-risk group that were stratified on the basis of their respective risk score signature. Univariate and multivariate Cox analyses of survival were conducted for the risk score signature and clinic-pathologic factors. The R package “survival” was used to perform the survival analysis.

### Constructing the nomogram

R software with the “rms” package was used to generate the nomogram. The concordance index (C-index) of the nomogram was generated from multivariate Cox regression analysis of all patients, with larger C-index indicating more accurate predictions. The total score for each patient was calculated through the generated nomogram, which was used to predict the rate of the 1-, 2-, 3-, and 5-year survival.

### Cell culture

U2OS cells were purchased from iCell (cat. no. iCell-h218) and cultured in McCoy’s 5A (Procell, cat. no. PM150710) medium containing 10% fetal bovine serum (FBS) and 1% antibiotics. 143-B cells were gifts from research associate Jianguo Feng and cultured in minimum essential medium (MEM) medium containing 10% FBS and 1% antibiotics. Cells were incubated in a 5% CO_2_ and 95% humidified incubator at 37°C.

### Q-PCR

Cells were collected for total RNA extraction according to the protocol of a total RNA extraction kit (TIANGEN, cat. no. DP419). Then, we used the Hifair III 1st Strand cDNA Synthesis Supermix for qPCR (gDNA digester plus) (YEASEN, cat. no. 11141ES60) to get cDNA of the relative cells. After getting cDNA, we used the Hieff qPCR SYBR Green Master Mix (High Rox Plus) (YEASEN, cat. no. 11203ES08) to do qPCR. in the StepOnePlus Real-Time PCR system. The primers used for qPCR were as follows:

GAPDH-F, 5′-GGAGCGAGATCCCTCCAAAAT-3′
GAPDH-R, 5′-GGCTGTTGTCATACTTCTCATGG-3′
E-cad-F, 5′-GTGGTCAAAGAGCCCTTACTGC-3′
E-cad-R, 5′-ACCGCTTCCTTCATAGTCAAACA-3′
VIM-F, 5′-TGCCGTTGAAGCTGCTAACTA-3′
VIM-R, 5′-CCAGAGGGAGTGAATCCAGATTA-3′
AIM2-F, 5′-ATAGCGCCTCACGTGTGTTA-3′
AIM2-R, 5′-TCTGTTACCTTCTGGACTACAAACA-3′
PKIB-F, 5′-AGTCTGGGGTCGCCAATTTT-3′
PKIB-R, 5′-TTTGCATCTTCCTTCACGGAG-3′
RPL22L1-F, 5′-GGAAATTTTGAGCAATTTCTACGGG-3′
RPL22L1-R, 5′-GCAACCACTCGAAGCCAATC-3′
PDPN-F, 5′-GCTTGACAACTCTGGTGGCA-3′
PDPN-R, 5′-GTGGCGCTTGGACTTTGTTC-3′
GZMA-F, 5′-GCAGCTCACTGTAACTTGAACA-3′
GZMA-R, 5′-GGTTTCACATCGTCCCCCTT-3′
MDM2-F, 5′-TCCAGAGAGTCATGTGTTGAGG-3′
MDM2-R, 5′-GCCATGGACAATGCAACCAT-3′
Bcl2-F, 5′-CAGGATAACGGAGGCTGGGATG-3′
Bcl2-R, 5′-GACTTCACTTGTGGCCCAGAT-3′
BAX-F, 5′-AAACTGGTGCTCAAGGCCC-3′
BAX-R, 5′-GGGACATCAGTCGCTTCAGT-3′

### DNA construction

To induce lentivirus, PKIB gene fragments were inserted into pLenti-CMV-GFP vectors. The primers used for amplifying the gene fragments were PKIB-F: CGCGGATCCGCCACCATGAGGACAGATTCATCAAAAA (contains *Bam*HI cutting site) and PKIB-R: ACGCGTCGACTCATTTTTCTTCATTTTGAGGCTTT (contains *Sal*I cutting site). The 2×Hieff Canace^®^ Gold PCR Master Mix was used to amplify target gene fragments following the manufacturer’s instructions (Yeasen, cat. no. 10149ES01). *Bam*HI (Takara, cat. no. 1605) and *Sal*I (Takara, cat. no. 1636) restriction enzyme were used to digest pLenti-CMV-GFP vectors and PKIB gene fragments. T4 DNA ligase (Thermo Fisher, cat. no. EL0016) was used to ligate the pLenti-CMV and PKIB gene fragments. Ligation products were transfected into Stbl3 competent cells, and plasmid sequencing was performed to confirm the sequence of constructed plasmids.

### Lenti virus induction and infection

To induce the lentivirus, HEK293T cells were co-transfected with pLenti-CMV-PKIB or pLenti-CMV-GFP plasmids (13 μg), pCMV-dR8.2 (5 μg), and pCMV–VSV-G (5 μg) using Hieff Trans™ Liposomal Transfection Reagent (Yeasen, cat. no. 40802ES02) in 10-cm dish. The virus was collected and filtered with a 0.22-μm filter after 72 h.

For infection, 143B cells were cultured in six-well plate. In the following day, cells were changed with 1 ml of fresh culture medium and 1 ml of virus supernatant was added. Seventy-two hours later, MEM culture medium containing puromycin (1 μg/ml) was used to select the infected cells. The selection lasted for 1 week.

### Western blot

Cells were collected and lysed with radio immunoprecipitation assay (RIPA) buffer containing phosphatase inhibitor cocktail (Yeasen, cat. no. 20109ES05) for 30 min on ice. Total protein lysis was used to measure target proteins expression. The anti–E-cad antibody (cat. no. BF0219) was purchased from Affinity. The anti-VIM antibody (cat. no. D21H3), anti-Akt antibody (cat. no. 4685), and anti–p-Akt antibody (cat. no. 4060) were purchased from Cell Signaling Technology. The anti-glyceraldehyde-3-phosphate dehydrogenase (GAPDH) antibody (cat. no. 10494-1-AP) and the anti-AIM2 antibody (cat. no. 66902-1-Ig) were purchased from ProteinTech. The anti-PKIB antibody (cat. no. ab233521) was purchased from Abcam. Total protein of 35 µg was loaded into 10% SDS-PAGE gel and run for about 90 min at 120 V. The gel was transferred on the polyvinylidene fluoride (PVDF) membrane and then incubated with 5% bovine serum albumin (BSA) for 1 h at room temperature. Then, the primary antibodies were incubated at 4°C overnight. Next, the blots were washed three times with TBST for 10 min. Last, the secondary antibodies were incubated at room temperature for 1 h by slowly shaking. The images of blots were taken with a standard chemiluminescence.

### Wound-healing assay

To explore the migration ability of osteosarcoma cell lines, we performed the wound-healing assay. Cells were cultured in six-well plate at a cell density of about 95% the next day in culture medium without FBS. We then used 200-µl tips to make healing. Cells were washed three times with PBS to remove the detached cells, and images were taken at time point of 0 h. Twelve hours later, images were taken to record the migration. The migrated area was analyzed through ImageJ software.

### Transwell assay

143-B cells and 143-B–PKIB cells were infected with GFP. The Matrigel (BD, cat. no. 356234) was dissolved in 4°C freezer overnight. The Matrigel was diluted with MEM medium without FBS as 1:4. Then, 50 μl of diluted Matrigel was added into 24-well transwell (Corning, cat. no. 3422), and the plate was placed in the incubator for 2 h. The 143-B–GFP and 143-B–PKIB–GFP cells were digested by trypsin, washed with PBS, and resuspended with MEM medium without FBS. Cells were counted, and 5 × 10^4^ cells were seeded into the upper chamber of the transwell. MEM medium (600 μl) with FBS was added in the lower chamber of the transwell. The plate was placed in the incubator. Twelve hours later, the medium in upper chamber was discarded, and a cotton swab was used to clean the upper chamber. Images were taken through fluorescence microscope. Furthermore, cell numbers were analyzed through ImageJ.

### CCK-8 assay

To measure the proliferation of cells, CCK-8 assay was performed. A total 3000 cells were counted and seeded in each well of 96-well plate with 100 μl of culture medium. Each time point was repeated for the three wells. CCK-8 reagent (Yeasen, cat. no. 40203ES60) was used to measure the optical density (OD) value at 450 nm following the manufacturer’s instruction. Three independent experiments were performed.

### Statistical analysis

Two-tailed Student’s t-test with unequal variants was used for statistical analysis. P-values lower than 0.05 were considered statically significant. K-M curves were used to estimate distinct prognosis by log-rank test. All analyses were performed with R software (version 3.5.1). pROC and survival packages were downloaded from Bioconductor (https://bioconductor.org).

## Results

### Identification of differentially methylated and expressed genes

In the current study, we performed an integrated analysis of DNA methylation data and gene expression file to identify key epigenetic markers in osteosarcoma. Information on the methylation status of 19,496 genes and the expression status of 19,495 genes were downloaded from TARGET database for subsequent analysis. The workflow was shown in **Figure S1**. We performed the univariate cox analysis of the DNA methylation profile and different screen analyses of gene expression profile of metastatic and non-metastatic osteosarcoma, respectively. Combined analysis was performed to identify the intersection genes between differentially methylated genes and differentially expressed genes. Then, the multivariable cox analysis was performed to identify the hub genes that are related to the metastasis and survival of osteosarcoma. To develop the nomogram, we also conduct multivariable cox by combining clinical data (**Figure S1**).

**Figure 1:**
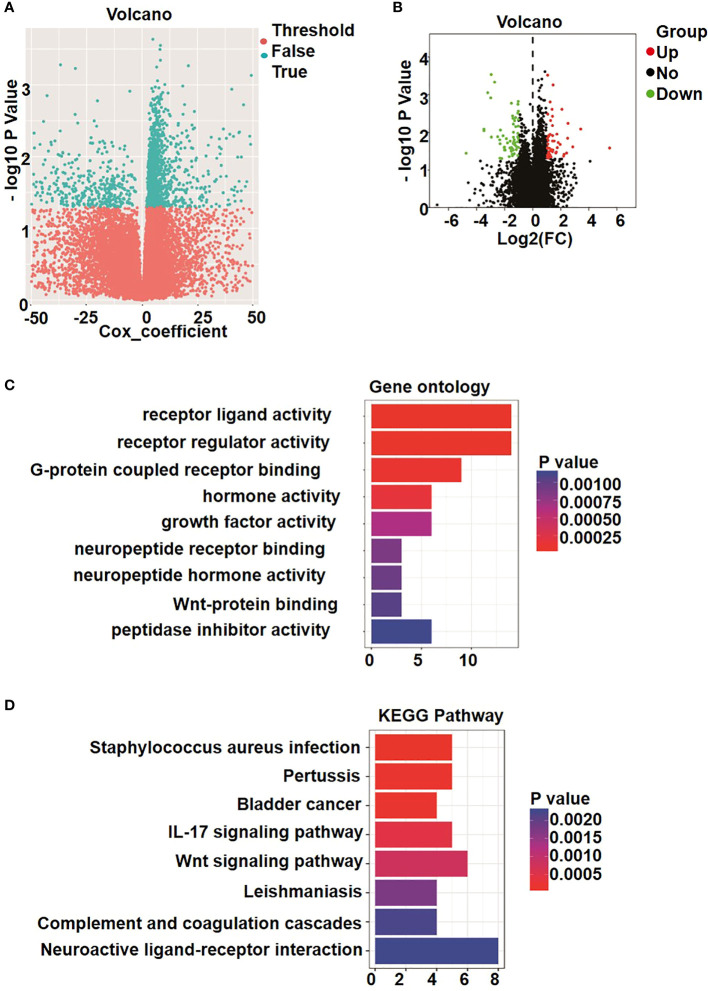
Identification of differently methylated and expressed genes. **(A)** Volcano plot shows the methylation data of 22 metastatic osteosarcoma and 64 non-metastatic osteosarcoma samples. A total of 1741 genes was considered to be significantly differently methylated, exhibiting in cyan dots. **(B)** Volcano plot of 147 genes in osteosarcoma patients. Blue color indicates up-regulated expression, and red color represents down-regulated genes. **(C)** Gene ontology (GO) enrichment analysis. **(D)** Kyoto Encyclopedia of Genes and Genomes analyses (KEGG) enrichment results.

Following quality control and normalization assessment to remove probes with SNP-CpG distance ≤2, tagging X and Y chromosomes, with minimum allelic frequency<0.05, and reduce cross hybridization, a total of 19,314 methylated genes remained in the final dataset of 86 osteosarcoma samples. Next, we performed univariate Cox proportional hazards regression analysis of the expression data of the compiled list of methylated genes. The analysis revealed 1,844 genes that were significantly correlated with OS of patients. Furthermore, 1,741 differentially methylated genes showed an absolute value of mean b-value difference of more than 0.01 (P< 0.05) between the metastatic and non-metastatic osteosarcoma groups (**Figure 1A**).

On the basis of the screening criteria P< 0.05 and |logFC| > 1, a total of 147 significant differentially expressed genes were identified among all metastatic and non-metastatic osteosarcoma samples. Overall, 61 genes were significantly upregulated (red dots in **Figure 1B**), and 82 genes were significantly downregulated (green dots in **Figure 1B**).

To evaluate the potential functions of these differentially expressed genes between patients with metastatic and non-metastatic osteosarcoma, we performed GO analysis that allows a comprehensive overview of their molecular functions, as well as involvement in biological process and localization. As shown in **Figure 1C**, the differentially expressed genes were mainly related to receptor ligand activity, receptor regulator activity, G protein–coupled receptor binding, and peptidase inhibitor activity and involved in regulating signaling transduction through cell membrane receptors. Kyoto encyclopedia of genes and genomes (KEGG) pathway enrichment analysis results further showed that the differentially expressed genes were enriched in signaling by neuroactive ligand–receptor interaction, Wnt signaling pathway, IL-17 signaling pathway, *Staphylococcus aureus* infection, and pertussis (**Figure 1D**). These signaling pathway activated by receptor-ligand binding, and the extracellular signals are transmitted to cells, regulating genes expression and affecting the proliferation and development of tumors (28, 29).

### Identification of intersection genes related to metastasis of patients with osteosarcoma

**Figure 2:**
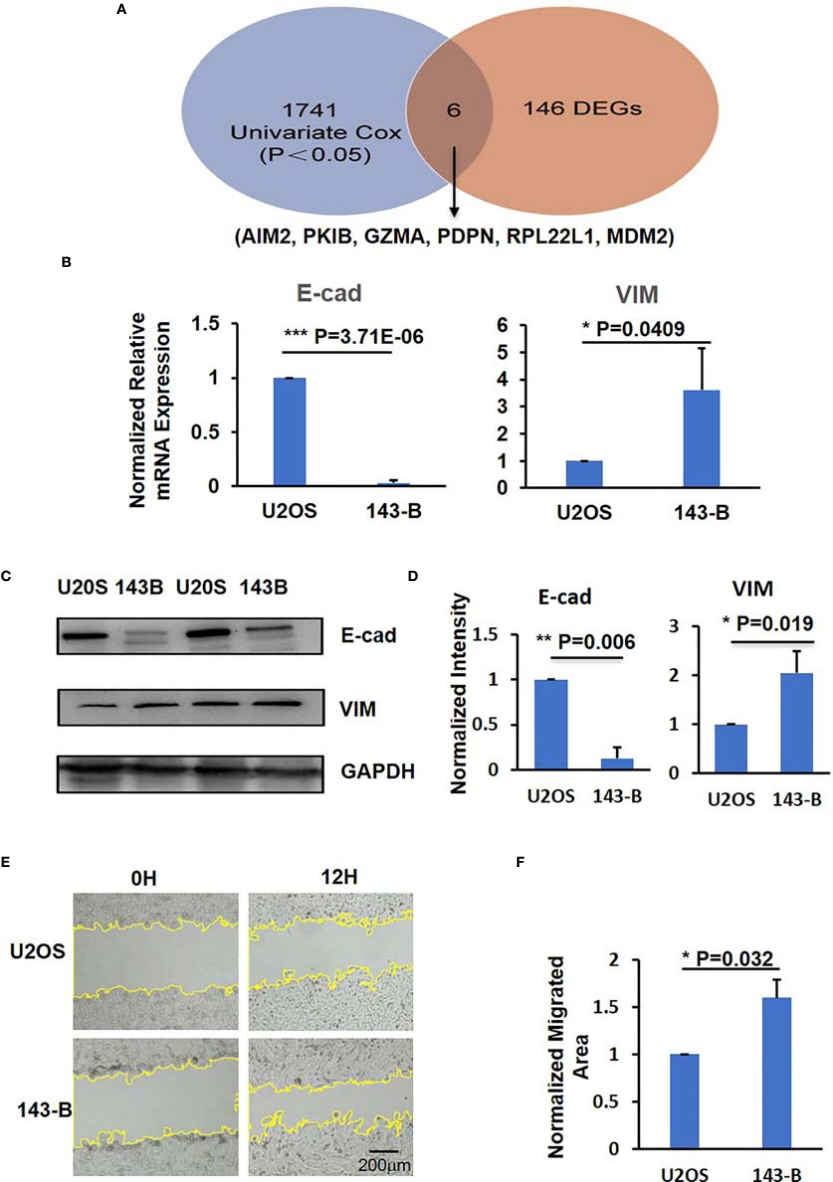
The identification of metastatic level of U2OS and 143-B osteosarcoma cells. **(A)** The intersection results of DEGs and survival-methylated genes. **(B)** Q-PCR results to show the expression level of E-cad and VIM. **(C)** Representative images of western blot show the protein expression level of E-cad and VIM. **(D)** Bar graphs showed the normalized protein expression intensity of E-cad and VIM. **(E)** Representative images show the wound-healing detected on time point 0h and 12h in U2OS and 143-B cells. **(F)** Bar graphs show the normalized migrated area of U2OS and 143-B cells as indicated in panel D. n>=3, Error bars: Mean±SD. *P<0.05, **P<0.01, ***P<0.001.

Further analysis on the differences between metastatic and non-metastatic samples revealed six intersection genes—*AIM2*, *RPL22L1*, *PDPN*, *PKIB*, *GZMA*, and *MDM2*—that related to metastasis potential of osteosarcoma and the observed gene expression differences resulted from the changes in methylation (**Figure 2A**). In addition, the gene ontology and KEGG pathways that these six identified intersection genes involved in were summarized in **Table 2**.

**Table 2:** The ontology and KEGG pathway that the hub genes are involved in.

Gene	Category	Term	Count	P-Value
**AIM2**				
	GOTERM_BP	GO:0006954~inflammatory response	8	0.0224
	GOTERM_BP	GO:0006915~apoptotic process	10	0.0230
	GOTERM_BP	GO:0071466~cellular response to xenobiotic stimulus	3	0.0724
	GOTERM_BP	GO:0035690~cellular response to drug	3	0.0763
**RPL22L1**				
	KEGG_PATHWAY	hsa05171: Coronavirus disease - COVID-19	6	0.0310
PDPN				
	GOTERM_BP	GO:0043066~negative regulation of apoptotic process	12	9.45E-04
	GOTERM_BP	GO:0003333~amino acid transmembrane transport	3	0.0249
	GOTERM_BP	GO:0008285~negative regulation of cell proliferation	7	0.0936
	GOTERM_CC	GO:0070161~anchoring junction	7	0.0889
	GOTERM_MF	GO:0005102~receptor binding	8	0.0173
	GOTERM_MF	GO:0051087~chaperone binding	4	0.0377
**PKIB**				
	GOTERM_BP	GO:0051973~positive regulation of telomerase activity	4	0.0016
**GZMA**				
	GOTERM_BP	GO:0043065~positive regulation of apoptotic process	8	0.0072
	GOTERM_BP	GO:0006915~apoptotic process	10	0.0230
	GOTERM_CC	GO:0005576~extracellular region	41	1.83E-10
	KEGG_PATHWAY	hsa04080: Neuroactive ligand–receptor interaction	9	0.0058
**MDM2**				
	GOTERM_BP	GO:0043066~negative regulation of apoptotic process	12	0.0009
	GOTERM_BP	GO:0042493~response to drug	8	0.0039
	GOTERM_BP	GO:0008284~positive regulation of cell proliferation	11	0.0043
	GOTERM_BP	GO:0045944~positive regulation of transcription from RNA polymerase II promoter	17	0.0075
	GOTERM_BP	GO:0071456~cellular response to hypoxia	5	0.0155
	GOTERM_BP	GO:0043278~response to morphine	3	0.0188
	GOTERM_BP	GO:0051726~regulation of cell cycle	7	0.0211
	GOTERM_BP	GO:0006915~apoptotic process	10	0.0230
	GOTERM_BP	GO:0002027~regulation of heart rate	3	0.0236
	GOTERM_BP	GO:0010628~positive regulation of gene expression	9	0.0241
	GOTERM_BP	GO:0072717~cellular response to actinomycin D	2	0.0269
	GOTERM_BP	GO:0071494~cellular response to UV-C	2	0.0467
	GOTERM_BP	GO:0003283~atrial septum development	2	0.0532
	GOTERM_BP	GO:0060411~cardiac septum morphogenesis	2	0.0849
	GOTERM_BP	GO:0065003~macromolecular complex assembly	4	0.0853
	GOTERM_MF	GO:0042975~peroxisome proliferator activated receptor binding	2	0.0775
	KEGG_PATHWAY	hsa05219: Bladder cancer	5	0.0002
	KEGG_PATHWAY	hsa05200: Pathways in cancer	13	0.0006
	KEGG_PATHWAY	hsa04151: PI3K-Akt signaling pathway	9	0.0051
	KEGG_PATHWAY	hsa04115: p53 signaling pathway	4	0.0178
	KEGG_PATHWAY	hsa04218: Cellular senescence	5	0.0305
	KEGG_PATHWAY	hsa05215: Prostate cancer	4	0.0372
	KEGG_PATHWAY	hsa05165: Human papillomavirus infection	7	0.0390
	KEGG_PATHWAY	hsa05202: Transcriptional misregulation in cancer	5	0.0586
	KEGG_PATHWAY	hsa04919: Thyroid hormone signaling pathway	4	0.0641
	KEGG_PATHWAY	hsa05169: Epstein–Barr virus infection	5	0.0671
	KEGG_PATHWAY	hsa05203: Viral carcinogenesis	5	0.0690
	KEGG_PATHWAY	hsa05205: Proteoglycans in cancer	5	0.0700
	KEGG_PATHWAY	hsa04110: Cell cycle	4	0.0706

To verify the expression level of the six intersection genes in metastatic and non-metastatic osteosarcoma, we compared the expression level of these six genes in U2OS and 143-B osteosarcoma cell lines, which represent the low metastatic and high metastatic osteosarcoma, respectively (30). Loss expression of E-cad was reported to associate with cancer metastasis, and VIM was reported to promote migration of cancer cells (31, 32). To confirm the migration and metastatic ability of U2OS and 143-B cells, we quantified the E-cad and VIM expression in these two cell lines. As shown in **Figure 2B**, compared with U2OS cell group, the expression of E-cad was downregulated and that of VIM was upregulated significantly in 143-B cells. The Western blot results showed the protein expression levels of E-cad and VIM, consistent with mRNA expression level (**Figures 2C, D**). Moreover, we also performed the wound-healing assay to compare the migration ability of U2OS and 143-B cells, and the results showed that the migration ability of 143-B was significantly higher than that of U2OS (**Figures 2E, F**). These results indicated that the 143-B has higher metastasis level than U2OS, which is consistent with the previous reports (30).

**Figure 3:**
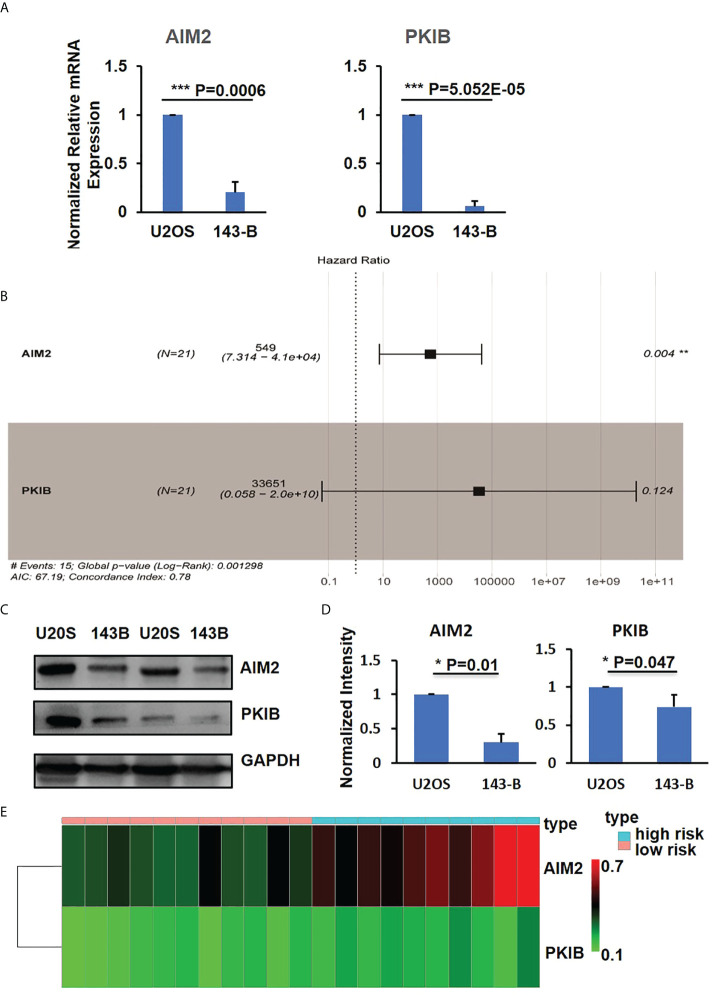
PKIB and AIM2 involved in survival of osteosarcoma. **(A)** Q-PCR results to show the expression level of AIM2 and PKIB genes. **(B)** Multivariate Cox regression analyses of AIM2 and PKIB genes with overall survival. **(C)** Representative images of western blot to show the protein expression level of AIM2 and PKIB. **(D)** Bar graphs showed the normalized protein expression intensity of AIM2 and PKIB. **(E)** The distribution of AIM2 and PKIB DNA methylation level. n>3, Error bars: Mean±SD. ***P<0.001.

We then measured the mRNA expression level of *PKIB*, *RPL22L1*, *PDPN*, *AIM2*, *GZMA*, and *MDM2* genes in U2OS and 143-B cells through Q-PCR. The expression of *PKIB*, *AIM2*, and *GZMA* was significantly decreased in high metastatic osteosarcoma 143-B cells compared with low metastatic osteosarcoma U2OS cells; the expression of *PDPN* and *RPL22L1* was higher in 143-B cells (**Figure 3A**, **Figure S2**); and the expression of *MDM2* was slightly increased in 143-B cells (**Figure S2**), which was consistent with the bioinformatics analysis results.

Focusing on the six intersection genes and their potential roles, we performed multivariable COX analysis and only identified two hub genes, *AIM2* and *PKIB* (**Figure 3B**), which were related to the metastasis of osteosarcoma. To further confirm the expression of AIM2 and PKIB in U2OS and 143-B cells, we performed the Western blot experiment. The results showed that AIM2 and PKIB were decreased in 143-B cells compared with that in U2OS cells (**Figures 3C, D**), which was consistent with gene expression results. In addition, the DNA methylation level of PKIB and AIM2 was higher in the high-risk samples compared with that in the low risk samples (**Figure 3E**), which suggests that the lower expression of these two genes in metastatic osteosarcoma was probably related to their DNA methylation level.

### *PKIB* involved in metastasis and proliferation of osteosarcoma

**Figure 4:**
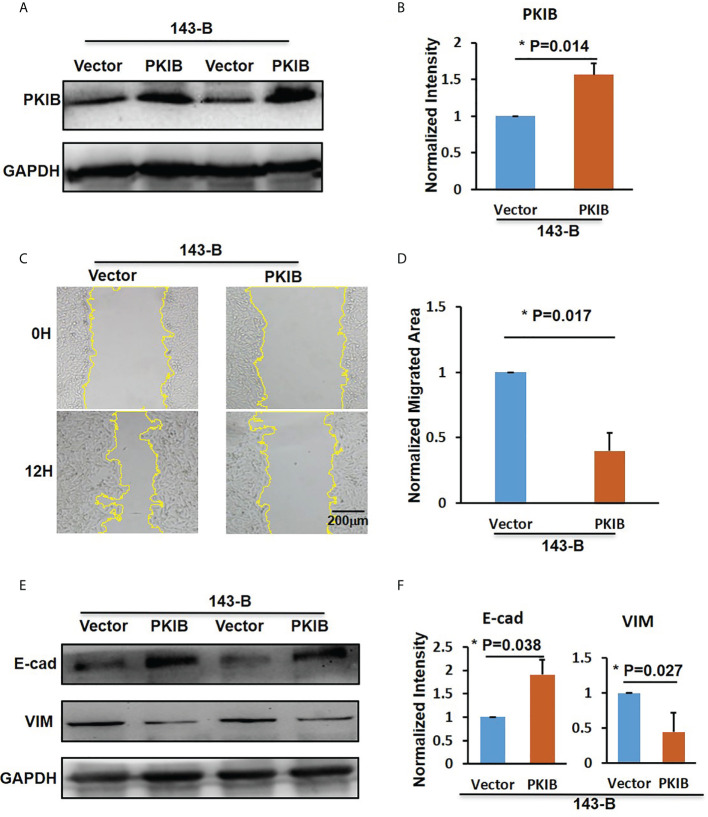
PKIB inhibit the migration of osteosarcoma. **(A)** Representative images of western blot to show the protein expression level of PKIB. **(B)** Bar graphs showed the normalized intensity of protein expression of PKIB indicated as in panel A. **(C)** Representative images show the wound-healing detected on time point 0h and 12h in 143-B cells with or without recombinant expression of PKIB. **(D)** Bar graphs show the normalized migrated area of 143-B cells recombinant express PKIB or not as indicated in panel C. **(E)** Representative images of western blot to show the protein expression level of E-cad and VIM. **(F)** Bar graphs showed the normalized intensity of protein expression of E-cad and VIM as indicated in panel E. n>=3, Error bars: Mean±SD, *P<0.05.

**Figure 5:**
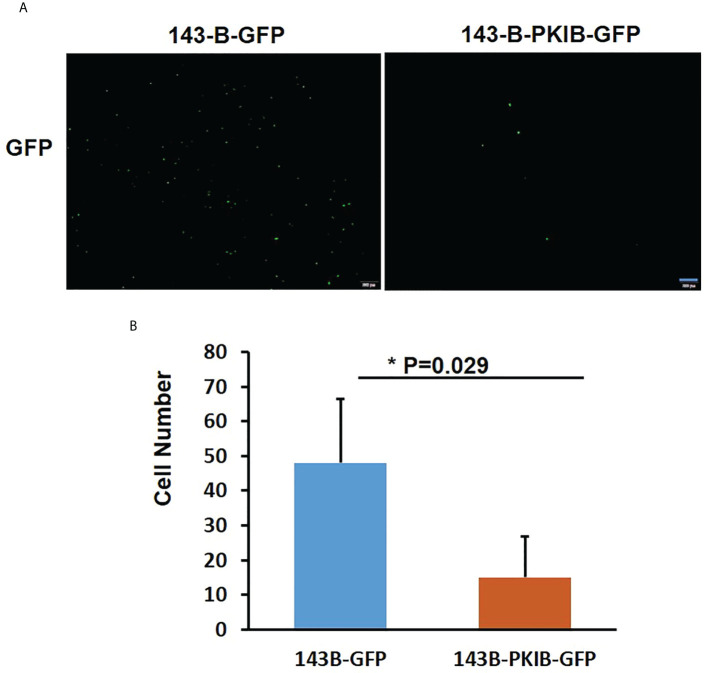
PKIB inhibits the invasion of osteosarcoma. **(A)** Representative images to show the 143-B-GFP and 143-B-PKIB-GFP cells invaded into lower chamber 12h after seeding on the upper chamber of transwell. **(B)**. Bar graph to show the average cell number invaded into lower chamber of transwell. n=4, Error bars: Mean±SD, *P<0.05.

In the previous results (**Figure 3B**), we could find that the *p*-value of PKIB hub gene was 0.124, which represented “not significant”; however, does it really has no meaning? To verify whether PKIB was related to the metastasis and survival of osteosarcoma as a hub gene, we performed several experiments. First, we recombinantly express the PKIB in 143-B cells by lenti-virus infection, and the Western blot results showed that the PKIB was successfully expressed in 143-B cells (**Figures 4A, B**). Then, the wound-healing assay was performed. As shown in **Figures 4C, D**, compared with 143-B cells infected with pLenti-CMV-GFP vector group, overexpressing the PKIB significantly inhibited the migration of 143-B cells. Furthermore, the Western blot showed that the protein expression of E-cad was increased, and the protein expression of VIM was decreased in 143-B cell recombinant expression PKIB (**Figures 4E, F**). To explore the effects of PKIB on the invasion of metastatic osteosarcoma, we performed transwell assay. 143-B cells and 143-B–PKIB cells were infected with GFP to label cells. As shown in **Figure S3**, the 143-B cells and 143-B–PKIB cells were successfully infected with GFP. Compared with 143-B–GFP group, the cell number of the 143-B–PKIB–GFP cells invaded into the lower chamber of transwell was significantly decreased (**Figures 5A, B**), indicating that PKIB inhibited the invasion of metastatic osteosarcoma cells. These results indicated that PKIB inhibited the metastasis of osteosarcoma.

**Figure 6:**
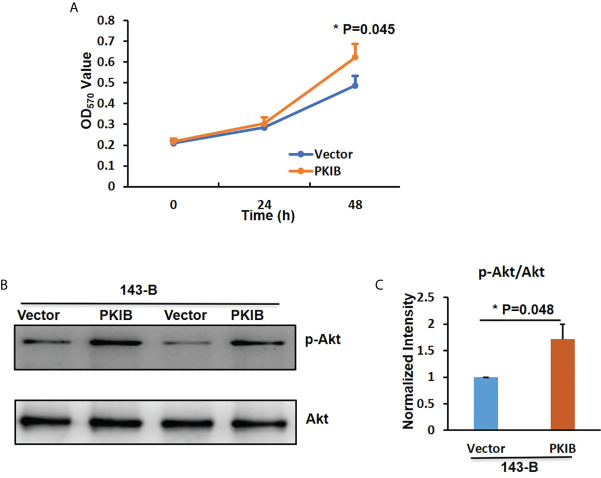
PKIB promotes the proliferation of osteosarcoma. **(A)** The OD450nm value to show the proliferation ability of 143B cells with or without PKIB overexpression as indicated. **(B)** Representative images of western blot to show the protein expression level of p-Akt and Akt. **(C)** Bar graphs showed the normalized intensity of p-Akt as indicated in panel B. n>=3, Error bars: Mean±SD, *P<0.05.

To explore the effects of PKIB on the proliferation of metastatic osteosarcoma cells, the CCK-8 assay was performed. As shown in **Figure 6A**, 143-B cells with recombinant expression of PKIB showed higher proliferation ability than control group. Recently, PKIB was reported involved in regulating proliferation of non–small cell lung cancer through PI3K/Akt signaling pathway (33). To further explore whether PKIB regulating the proliferation of osteosarcoma cells through PI3K/Akt signaling pathway as well, we performed the Western blot to measure the activation level of Akt. As shown in **Figure 6B, C**, the level of phosphorylated Akt was higher in 143-B–PKIB group than that in control group, indicating that overexpressing PKIB activated the Akt signaling pathway and may involve in proliferation regulation. These results showed that PKIB plays an important role in regulating the proliferation, migration, and even metastasis of osteosarcoma, giving us clues that PKIB could be a potential treatment target for patients with metastatic osteosarcoma.

### Identification of *AIM2*- and *PKIB*-related signature in patients with osteosarcoma

On the basis of the previous results, we found that the DNA methylation levels of these two genes were related to the risk-score, with a high DNA methylation level of *AIM2* and *PKIB* being associated with high risk, and *AIM2* and *PKIB* were involved in the OS of patients with osteosarcoma. Thus, hub genes of *AIM2* and *PKIB* were selected to construct the risk signature for osteosarcoma. To ease the use of *AIM2*/*PKIB* as prognostic tool in routine clinical practice, the following formula was developed to generate a risk score for each patient:

RS=(6.31×*ev*_*AIM*2_)+(10.42×*ev*_PKIB_), in which *ev* represents the gene expression value.

**Figure 7:**
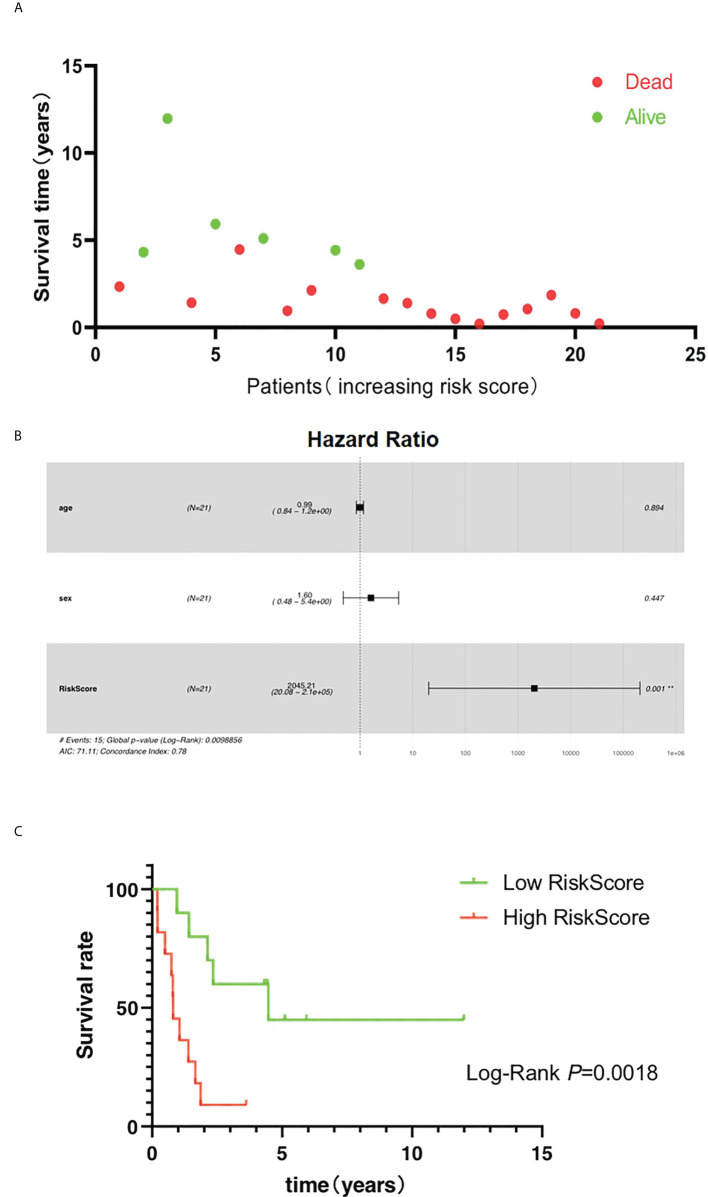
Risk score is the major clinical factors related to overall survival of osteosarcoma patients. **(A)** The distribution of survival time. **(B)** Multivariate Cox regression analyses of clinical factors with overall survival. **(C)** The K-M plot of the high risk and low risk groups.

Higher risk score was found to help predict a shorter survival time of patients (**Figure 7A**). Moreover, the results showed that the OS rate was significantly related to the risk score but not to gender or age of the patients (**Figure 7B**, **Table 1**). Patients with high-risk score were found to be more likely to experience shorter OS (**Figure 7C**). These results indicate that *AIM2*/*PKIB*-based risk score could be considered as a valuable tool to predict OS rate in patients with osteosarcoma.

### Construction of nomogram for osteosarcoma

**Figure 8:**
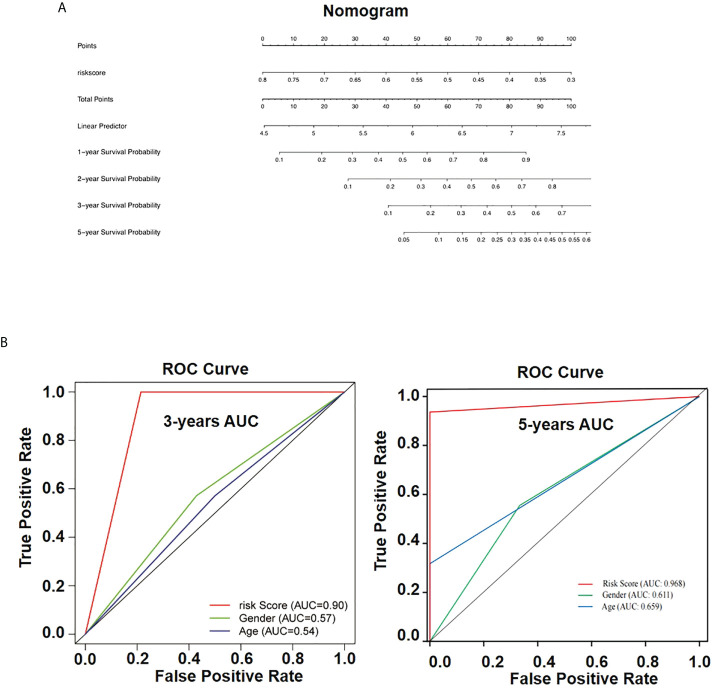
Nomogram for overall survival prediction in osteosarcoma patients. **(A)** The nomogram consists of risk score based on the AIM2/PKIB expression profile. **(B)** The ROC curves of the risk subgroups by the tertiles of total points derived from the nomogram.

To generate a valuable tool to predict the OS rate of patients with osteosarcoma, we further analyzed clinical data and build a nomogram based on risk scores of patients with osteosarcoma that were combined with the identified prognostic signature (**Figure 8A**). According to the model obtained, we could predict the 1-, 2-, 3-, and 5-year survival rates of patients with expression patterns of *AIM2* and *PKIB*. OS nomogram-predicted rates were similar to the best prediction performance. Receiver operating characteristic (ROC) analysis results showed that the area under the ROC curve for the 3-year and 5 year-survival was 0.90 and 0.968 for the risk score, respectively (**Figure 8B**). These findings further demonstrate that the *AIM2*/*PKIB* expression–based risk score signature has great predictive accuracy compared with common clinical factors.

## Discussion

Strategies for osteosarcoma treatment vary from chemotherapy, radiotherapy, and surgery to immunotherapy; however, the OS rate is still low (34, 35). Systemic metastasis is still the major reason for poor prognosis in patients with osteosarcoma, with approximately 20% of cases being reported metastatic at diagnosis (6, 36). The lung is the organ that is most frequently affected by metastasis, with pleural metastasis being also reported (37). The underlying mechanism of osteosarcoma metastasis has been an important research topic. Indeed, benefiting from recent bioinformatics development, recent discoveries have shed some light into mechanisms involved in osteosarcoma metastasis. Tian et al. (38) used weighted gene co-expression network analysis (WGCNA) to identify several osteosarcoma metastasis–associated genes—*IGFBP5*, *IGFBP6*, *WISP3*, and *MYL2*—which were, in turn, found to be involved in insulin-like growth factor–binding pathway. Wang et al. (39) also used WGCNA methods to identify co-expression modules and pathways related to osteosarcoma metastasis. In this study, we used combined analysis of methylation profile and expression profile to identified six intersection genes—*AIM2*, *RPL22L1*, *PDPN*, *PKIB*, *GZMA*, and *MDM2*, which was related to the metastasis of patients. In addition, further analysis together with clinical data showed that *AIM2* and *PKIB* were identified as hub genes related to OS of patients with osteosarcoma.

AIM2 is induced by interferon-gamma and plays a crucial role in human solid tumors as a tumor suppressor gene. Previous studies showed that AIM2 can prevent metastasis development of human renal carcinoma by promoting autophagy (40), whereas loss of AIM2 mediated by HBx can promote hepatocellular carcinoma metastasis (41). In addition, low expression of AIM2 combined with high levels of phosphorylated STAT3 is predictive of poor prognosis in hypopharyngeal squamous cell cancer (42). Dihlmann et al. (43) reported that up to 67.4% of colorectal cancers have reduced AIM2 expression, which can be used as biomarker of poor prognosis. Methylation of *AIM2* locus was previously reported to be related to trauma exposure, post-traumatic stress disorder, and C-reactive protein associations (44). Moreover, evidence suggests that development of type 2 diabetes mellitus may be supported by reduced total methylation of *AIM2* promoter region (45). We also found that *AIM2* was highly methylated in patients with high-risk osteosarcoma and was expressed lower in metastatic osteosarcoma cells, which was consistent with the point that loss of *AIM2* promotes cancer metastasis (40, 41).

The cAMP-dependent PKIB has been shown to contribute for tumor progression. Reports suggest that PKIB can promote proliferation and metastasis of NSCLC cells, as well as support malignant transformation of triple-negative breast cancer, promote infiltration of colorectal carcinoma, and support prostate cancer aggressiveness (33, 46–48). Here, we reported that PKIB promotes the proliferation of high metastatic osteosarcoma cells through Akt signaling pathway. Furthermore, we found that PKIB significantly inhibited the migration and metastasis of high metastatic osteosarcoma cells, indicating that PKIB plays important roles in the process of metastasis of osteosarcoma and could be a potential target for osteosarcoma treatment. However, the mechanism of PKIB regulated the metastasis of osteosarcoma is still unclear.

Prognosis assessment is of great significance in oncology and medicine. Medical nomogram, which is a statistical prognostic model graphically depicting biologic and clinical variables to generate the probability of a clinical event, is a valuable and meaningful tool for predicting survival of patients (49, 50). Nowadays, nomograms constructed on the basis of information of genes, microRNAs, or long non-coding RNAs are widely used to improve prognosis prediction for several types of cancers, including esophageal squamous cell carcinoma (51). Mao et al. (52) developed a nomogram to predict the survival of stage IIIA-N2 non–small cell lung cancer after surgery, in which positive lymph node, the lymph node examined, tumor size, and age, among other clinical factors, were identified as significant prognostic variables. A nomogram that could estimate the 3- and 5-year overall and cancer-specific survival of high-grade osteosarcoma has been developed on the basis of age, primary site, and tumor size (53). In this study, we developed a new nomogram to predict the 1-, 2-, 3-, and 5-year OS for metastatic osteosarcoma based on *AIM2* and *PKIB* expression–related risk score. The proposed nomogram showed to be easy to use by clinicians while retrieving high prognostic accuracy.

In conclusion, we combined analyzed the DNA methylation profile and gene expression profile in metastatic and non-metastatic osteosarcoma cells and screened for differentially methylated and differentially expressed genes. With this approach, we identified expression of *AIM2* and *PKIB* as being valuable tools to assess risk in patients with osteosarcoma. In addition, the experimental results showed that PKIB significantly inhibits the migration and promotes the proliferation of metastatic osteosarcoma cells through affecting the phosphorylation level of Akt. Finally, we constructed a nomogram on the basis of *AIM2*/*PKIB* expression–based risk score to predict prognosis of the 1-, 2-, 3-, and 5-year OS of patients with osteosarcoma, offering clinical value for prognosis prediction and support osteosarcoma treatment decision process.

## Data availability statement

The original contributions presented in the study are included in the article/**Supplementary Material**. Further inquiries can be directed to the corresponding authors.

## Ethics statement

Ethical review and approval was not required for the study on human participants in accordance with the local legislation and institutional requirements. Written informed consent from the patients/participants or patients/participants’ legal guardian/next of kin was not required to participate in this study in accordance with the national legislation and the institutional requirements.

## Author contributions

RW, GY, QL, XF, ZL, HM, HL, and WH conceived the project. RW, QL, XF, and ZL performed the experiments. RW and GY analyzed data and wrote the manuscript. HM, HL, and WH wrote and revised the manuscript. All authors contributed to the article and approved the submitted version.

## Funding

This research was funded by the Guangdong Basic and Applied Basic Research Foundation (No. 2019A1515011854, to RW), China Postdoctoral Science Foundation (No. 2020M672561, to RW), Sichuan Applied Basic Research Project (2018JY0402, to HL), Luzhou Municipal People’s Government–Southwest Medical University Science and Technology Strategic Cooperation Project (2018LZXNYD-ZK19, to HL), Sanming Project of Medicine in Shenzhen (SZSM201612019, to WH), and National Natural Science Foundation of China (No. 61427807, to WH).

## Acknowledgments

The authors thank Shuai Hong for doing the proofread.

## Conflict of interest

The authors declare that the research was conducted in the absence of any commercial or financial relationships that could be construed as a potential conflict of interest.

## Publisher’s note

All claims expressed in this article are solely those of the authors and do not necessarily represent those of their affiliated organizations, or those of the publisher, the editors and the reviewers. Any product that may be evaluated in this article, or claim that may be made by its manufacturer, is not guaranteed or endorsed by the publisher.

## Supplementary material

Figure S1 Workflow of the study.

Figure S2 The expression level of intersection genes in U2OS and 143-B cells. Q-PCR results to show the expression level of *GZMA, PDPN, RPL22L1*, and *MDM2* genes.

Figure S3 The recombinant expression of GFP in 143-B and 143-B-PKIB cells. Representative images to show the GFP channel and DIC channel of 143-B and 143-B-PKIB cells.

Figure S4 The expression of apoptosis related genes in 143-B cells with or without recombinant expression of PKIB. Q-PCR results to show the gene expression of Bcl-2 and BAX.
