# Supplementary figures and images for: PKIB involved in the metastasis and survival of osteosarcoma
Source: Front Oncol. 2022 Aug 22;12:965838. doi: 10.3389/fonc.2022.965838 (PMC9441607; doi:10.3389/fonc.2022.965838)

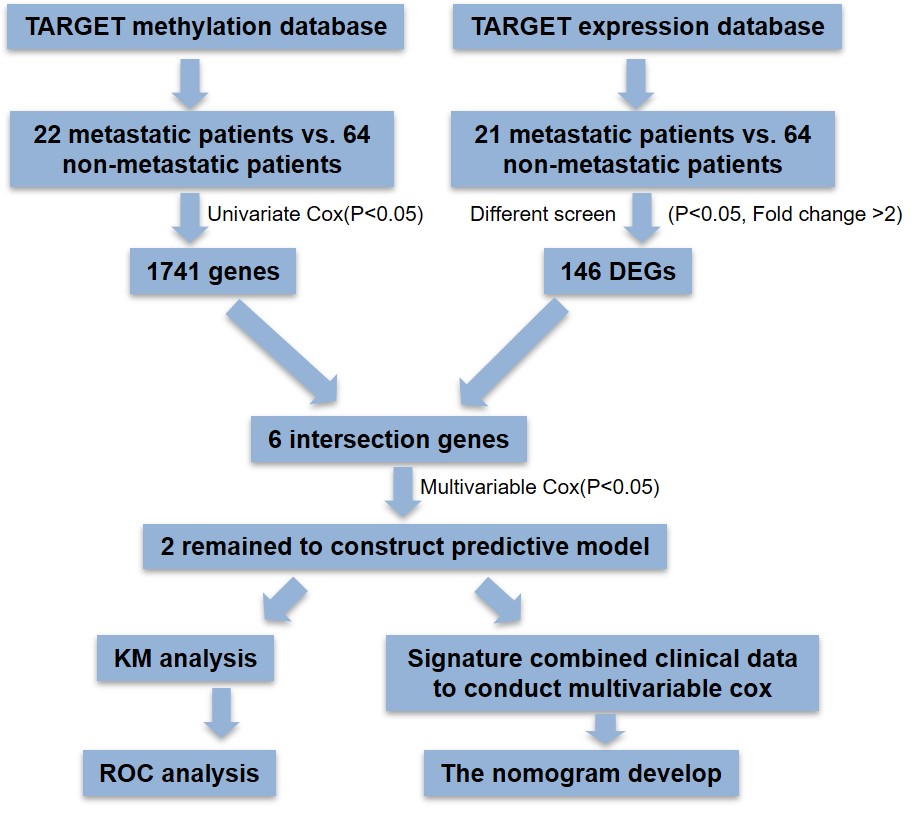

Supplement: Figure S1 — Workflow of the study. [file Image_1.jpeg]

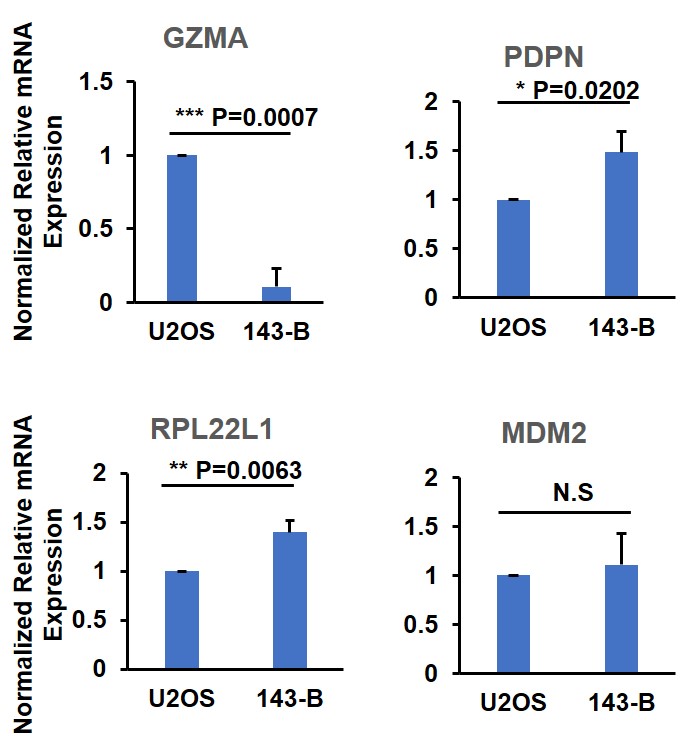

Supplement: Figure S2 — The expression level of intersection genes in U2OS and 143-B cells. Q-PCR results to show the expression level of GZMA, PDPN, RPL22L1, and MDM2 genes. [file Image_2.jpeg]

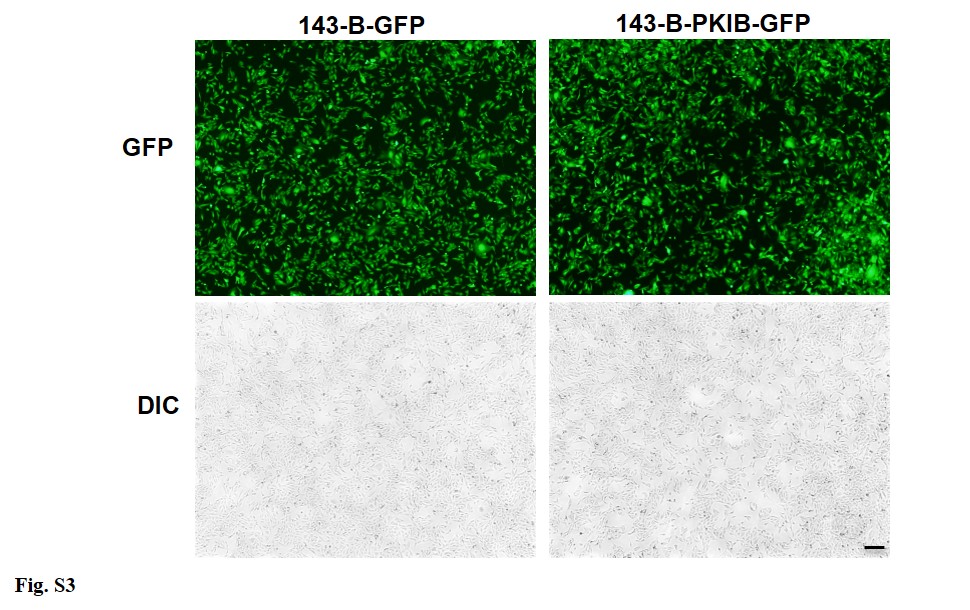

Supplement: Figure S3 — The recombinant expression of GFP in 143-B and 143-B-PKIB cells. Representative images to show the GFP channel and DIC channel of 143-B and 143-B-PKIB cells. [file Image_3.jpeg]

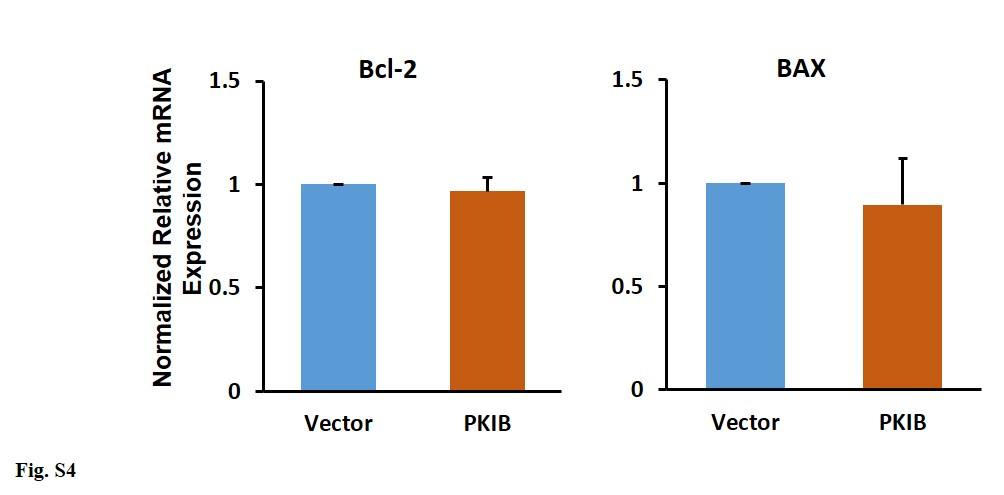

Supplement: Figure S4 — The expression of apoptosis related genes in 143-B cells with or without recombinant expression of PKIB. Q-PCR results to show the gene expression of Bcl-2 and BAX. [file Image_4.jpeg]
